# Metabolite profile data of grapevine plants with brown wood streaking and grapevine leaf stripe (esca complex disease) symptoms

**DOI:** 10.1016/j.dib.2021.107623

**Published:** 2021-11-20

**Authors:** Piebiep Goufo, Isabel Cortez

**Affiliations:** a Centre for the Research and Technology of Agro-Environment and Biological Sciences, Universidade de Trás-os-Montes e Alto Douro, Quinta de Prados, Vila Real 5000-801, Portugal; b Departamento de Agronomia, Universidade de Trás-os-Montes e Alto Douro, Quinta de Prados, Vila Real 5000-801, Portugal

**Keywords:** Grapevine trunk diseases, Metabolomics, *Fomitiporia mediterranea*, Metabolic profiling, Esca complex disease, *Phaeomoniella chlamydospora*, *Phaeoacremonium minimun*, Disease progression, ASY, asymptomatic leaves, SY1, chlorotic leaves, CTL, healthy control vines, SY2, scorched and spotted leaves, UHPLC, ultrahigh performance liquid chromatography, HRaMS, high resolution/accurate mass spectrometer, HESI-II, heated electrospray ionization source, LIMS, Metabolon's laboratory information management system, RSDs, relative standard deviations

## Abstract

Leaf samples were obtained from *Vitis vinifera* ‘Malvasia Fina’ plants with well-characterized esca complex disease symptoms (*n* = 18) and from healthy uninfected plants (*n* = 6). Leaves from diseased plants were divided into three groups: asymptomatic (ASY), chlorotic (SY1), and scorched leaves (SY2). The metabolic profile of these leaves was then examined using an ultrahigh performance liquid chromatography system coupled to a Q-Exactive Hybrid Quadrupole-Orbitrap high resolution/accurate mass spectrometer interfaced with a heated electrospray ionization source. The number of small molecules measured in a sample was increased by varying the reconstitution solvent, chromatographic column, and ionization source. Data on accurate masses, peak areas, and relative levels of several metabolites were documented for each leaf sample, using the abovementioned approach. In this paper, data on 235 metabolites of known structural identity are reported, along with the biochemical pathways to which the metabolites belong. The remaining data related to lipid species and with a different focus of the research question are reported elsewhere. The broad coverage of metabolites reported here resulted in a greater coverage of the biochemical pathways involved in grapevine metabolism, which could provide a better understanding of the biochemical changes occurring during the onset and progression of foliar symptoms after invasion of woods by esca-associated pathogens. To determine which metabolites varied according to the study design, the detected ion features were processed using different statistical methods, including mean and median values, fold changes, Welch's two-sample t-test, false discovery rate, and quartiles represented by box and whisker plots. The goal of this statistical evaluation was to assess the responses of healthy, asymptomatic, and symptomatic leaf groups using a pairwise comparison, thus providing an opportunity for detecting statistically significant compounds and uncovering the dynamic metabolic models underlying disease latency and symptom expression.

## Specifications Table

**Table:** 

Subject	Biological sciences (Omics: Metabolomics)
Specific subject area	Metabolomics as a tool for studying plant–pathogen interactions, including perturbations to primary metabolism and signaling and output pathways.
Type of data	Table
How the data were acquired	Metabolites were extracted from leaf samples with methanol, using an automated liquid handling robot (MicroLab STAR®, Hamilton Robotics Inc., Reno, NV, US). Metabolite separation was performed using Ultrahigh performance liquid chromatography (ACQUITY UHPLC System, Waters Corporation, Milford, MA, US). Accurate masses were obtained using a Q-Exactive Hybrid Quadrupole-Orbitrap high resolution/accurate mass spectrometer (HRaMS) interfaced with a heated electrospray ionization (HESI-II) source (Thermo Fisher Scientific, Waltham, MA, US). ThermoFisher Scientific (Waltham, MA, US) software Xcalibur QuanBrowser 3.0 was used for initial peak extraction and processing. Peak were identified using the Laboratory Information Management System (LIMS, Metabolon, Inc, Morrisville, NC, US). Standard statistical analyses were performed in ArrayStudio 10.0 (OmicSoft Corporation, Cary, NC, US).
Data format	Raw Analyzed
Description of data collection	After six years of consecutive annual inspections, vines (*Vitis vinifera* ‘Malvasia Fina’) with wood and/or foliar symptoms of esca complex disease were identified in an experimental vineyard. Six biological replicates of healthy leaves, asymptomatic leaves, symptomatic leaves with chlorosis, and symptomatic leaves with scorch and spots were collected from unaffected and diseased vines.
Data source location	Institution: Universidade de Trás-os-Montes e Alto Douro City/Town/Region: Quinta de Nossa Senhora de Lourdes, Vila Real Country: Portugal Latitude and longitude for collected samples/data: 41° 17.12′ 31′′ N, 7° 44.07′ 22′′ W, 465 m
Data accessibility	Repository name for metabolites: MENDELEY DATA Data identification number: 10.17632/r22wypf38w.1 (Metabolome of leaves of esca-affected grapevine) Direct URL to data: https://data.mendeley.com/datasets/r22wypf38w/1 Repository name for spectra: METABOLIGHTS Data identification number: MTBLS353 (Mass spectral metabolomic data of biochemical compounds in vine plants colonized by grapevine trunk diseases pathogens (esca complex disease)) Direct URL to data: https://www.ebi.ac.uk/metabolights/MTBLS353

**Value of the Data**

• The principal goal of the data provided in this paper is to allow a comprehensive understanding of the physiological processes occurring in plants in response to wood infection by fungi associated with grapevine trunk diseases and to discover new insights into specific biological phenomena such as the delayed appearance of foliar symptoms and yearly discontinuity in foliar symptom expression.
• The metabolome data can be useful to researchers working on trunk diseases, while the spectra data is of great interest to the OMIC research community. Recently, some leaf and wood OMIC data of vines with grapevine trunk diseases have been published [1], [2], [3], [4], [5], [6]. By correlating all data, researchers might retrieve important findings on the ability of this plant to slow down colonization by fungi.
• Furthermore, the analysis of these leaf data might strengthen or refute hypotheses that have been formulated related to grapevine trunk diseases, for example, the hypothesis that considers the translocation of toxic fungal metabolites from woody tissues to leaves through the phloem stream to explain foliar symptom appearance [1].
• The non-targeted metabolomics data reported here may also be used directly to identify potential biomarkers for early disease diagnosis (metabolites differentiating healthy and asymptomatic vines), followed by targeted metabolomics to validate the biomarkers in subsequent studies, based on standard analytical chemistry techniques.

## 1 Data Description

Twenty-four grapevine leaf samples were subjected to metabolomic analyses (SAMPLE_NUMBER 1 to 24 in Tables S1 and S2 in the Mendeley repository). The original source identifier grouped the samples (Group _NUMBER) into 1 = control (no signs of symptoms and no pathogen in the canes, cordon, and trunk); 2 = asymptomatic (presence of wood symptoms, i.e., wood streaking, but no foliar symptoms); 3 = symptomatic_level_1 (leaves with typical esca chlorosis, but no necrotic lesions); and 4 = symptomatic_level_2 (chlorotic spots between the leaf veins or along the leaf margins). There were six biological replicates for each of the four leaf groups. In order to facilitate sample handling and result analyses (STATISTICAL_COMPARISONS), a new designation was created for each sample received, for example, CTL-01 for replicate 1 of the Control group, ASY-04 for replicate 4 of the asymptomatic group, SY1-06 for replicate 6 of the Symptomatic_level_1 group, and SY2-2 for replicate e of the Symptomatic_level_2 group (Tables S1 and S2).

The present dataset comprises a total of 235 metabolites of known identity detected in the positive and negative injection modes (Tables S1–S3). Most of the metabolites were quantified using LC/MS positive ion monitoring for acidic compounds, followed by LC/MS negative ion monitoring for polar compounds, and LC/MS negative ion monitoring for basic compounds (Table 1). Each metabolite was assigned its own number (Compound number 1 to 235), along with the Metabolon's Laboratory Information Management System (LIMS) ID (Tables S1–S4). The identification of metabolites was based in part on the comparison of their retention index (RI) and accurate masses provided in Tables S1–S3. Other criteria for identification are described in the Methods section. The metabolomic dataset was based on the same experiment and methodology that yielded the lipidomic dataset previously reported by Goufo and Cortez [8]. Since metabolomic and lipidomic each has a different focus, segmenting the whole data into lipidomic and metabolomic datasets was done with the aim of studying the data from completely different angles. There is no overlap in the two datasets. Reference spectra and structures for the 235 metabolites were deposited in the repository Metabolights with accession number MTBLS353 [7], along with the spectra of 158 lipid species in Goufo and Cortez [8]. The percentage of replicates in which a metabolite was detected is provided for each leaf group in Table S3 (% Detected by Group in RAW data) and varied from 0% for a few metabolites to 100% for most metabolites.

**Table 1:** Distribution of the 235 identified metabolites per detection method, biochemical family and sub-pathways

Detection platform	Number of metabolites	Biochemical family	Number of metabolites	Sub-pathway	Number of metabolites
LC/MS Acidic	138	Amino Acid	118	Glutamate family (alpha-ketoglutarate derived)	40
LC/MS Polar	77	Carbohydrate	58	Aspartate family (oxaloacetate/aspartate derived)	23
LC/MS Basic	20	Cofactors, Prosthetic Groups, Electron Carriers	20	Amino sugar and nucleotide sugar	20
		Nucleotide	31	Branched Chain Amino Acids	18
		Peptide	8	Sucrose, glucose, fructose metabolism	17
				Pyrimidine metabolism	16
				Purine metabolism	15
				Aromatic amino acid metabolism (phosphoenolpyruvate derived)	13
				Glutathione metabolism	10
				Serine family (phosphoglycerate derived)	9
				Tricarboxylic acid (TCA) cycle	9
				Nicotinate and nicotinamide metabolism	8
				Vitamin B metabolism	8
				Dipeptide	8
				Glycolysis	6
				Amines and polyamines	5
				Ascorbate metabolism	4
				Calvin cycle and pentose phosphate	3
				Photorespiration	3

Empirical names (METABOLITE NAME) and chemical structures (SMILES = Simplified Molecular-Input Line-Entry System) are provided for each given accurate mass (Tables S1–S4). Whenever possible, the metabolites were associated with the open chemical and biological databases ChemIDplus (https://chem.nlm.nih.gov/chemidplus/), CAS SciFinder (https://scifinder.cas.org), PubChem (https://pubchem.ncbi.nlm.nih.gov), ChemSpider (http://www.chemspider.com), Kyoto Encyclopedia of Genes and Genomes (KEGG, https://www.genome.jp/kegg/), Human Metabolome Database (HMDB, http://www.hmdb.ca/), and Plant Metabolic Pathway Databases (PLANT CYC, https://plantcyc.org/) (Tables S1–S3). Compounds for which a matching pure standard was not available for confirmation were denoted by adding an asterisk symbol after the name of the metabolite. However, a high confidence in the identity of the compound was provided by m/z and classical structural analyses. The identified compounds represented five biochemical families (labeled SUPER PATHWAYS in Tables S1–S4), including amino acids, carbohydrates, cofactors + prosthetic groups + electron carriers, nucleotides, and peptides (Table 1). These metabolites were grouped into 19 SUPERPATHWAYS in the dataset according to Metabolon Pathway orders (Metabolync™, https://www.metabolon.com/) (Table 1).

For each peak, the area under the curve to the baseline (area count) was used as the first measure of the amount of the compound. The area counts were normalized to the sample weight to obtain the raw data, which are labeled “LEVELS” in Table S1. Raw data were rescaled to set the median equal to 1, and scaled imputed data, shown in Table S2**,** were obtained. Scaled imputed data were used in statistical analyses to calculate the mean values, fold changes, *P*-values (Welch's two-sample t-tests), and q-values (Table S3). Statistical comparisons contrasted leaves from each group to those of the other groups. A pathway heatmap associated with fold-change values was generated using *P*-values for each comparison. The colors used in the heatmap to highlight the differences between the groups are explained in Table 2. A summary of the number of metabolites that achieved statistical significance is shown in Table 3.

**Table 2:** Colors used in the heatmap for determining whether the level of a metabolite was significantly increased or decreased.

Heatmap	Color	Significance	Fold change
	Green	Significant difference between the groups is clearly shown; *P*-value is less than or equal to alpha (0.05).	Metabolite ratio (test variable 1/test variable 2) of < 1.00 i.e., fold change for the metabolite is decreased.
	Light green	Statistical cutoff for significance is narrowly missed; *P*-value is close to alpha (between 0.05 and 0.1).	Metabolite ratio of < 1.00.e., fold change for the metabolite is decreased.
	Red	Significant difference between the groups is clearly shown; *P*-value is less than or equal to alpha (0.05).	Metabolite ratio (test variable 1/test variable 2) of ≥ 1.00i.e., fold change for the metabolite is increased.
	Light red	Statistical cutoff for significance is narrowly missed; *P*-value is close to alpha (between 0.05 and 0.1).	Metabolite ratio (test variable 1/test variable 2) of ≥ 1.00i.e., fold change for the metabolite is increased.
	Non-colored text and cell	Mean values are not statistically different for the comparison.	The direction of change of the fold change does not matter.

**Table 3:** Number of metabolites that showed increased (UP) or decreased (DOWN) levels in pairwise comparisons between leaf groups. CTL = control group; ASY = asymptomatic group; SY2 = symptomatic group with severity level 1; SY2 = symptomatic group with severity level 2. Sig = significance

Pairwise comparison	Sig Meet Criteria (p ≤ 0.05)	Sig Up|Down	Sig Meet Criteria (p < 0.10)	Sig Up|Down	Sig Meet Criteria (0.05 < p < 0.10)	Sig Up|Down
**ASY /CTL**	**45**	**12 | 33**	**64**	**18 | 46**	**17**	**6 | 11**
**SY1 / CTL**	**77**	**47 | 30**	**97**	**56 | 41**	**20**	**9 | 11**
**SY2 / CTL**	**134**	**62 | 72**	**149**	**64 | 85**	**15**	**2 | 13**
**SY1 / ASY**	**65**	**44 | 21**	**86**	**56 | 30**	**21**	**12 | 9**
**SY2 / ASY**	**127**	**68 | 59**	**147**	**76 | 71**	**20**	**8 | 12**
**SY2 / SY1**	**138**	**62 | 76**	**145**	**67 | 87**	**16**	**5 | 11**

In Table S4, box and whisker plots are provided for each metabolite detected, sorted by biochemical sub-pathways. The plots display not only the mean values (represented by the symbol plus) and median values (represented by the horizontal line within the box) for six replicates but also extreme data points. The upper and lower quartiles are represented by the top and lower edges of the box, respectively. The bars show the 75% (maximum) and 25% (minimum) percentiles across replicates.

## 2 Experimental Design, Materials and Methods

The data presented in this paper and that in Goufo and Cortez [8] were all collected within the same work, using the same samples and equipment. Thus sampling, sample preparation, metabolite extraction, chromatography-mass spectrometry and statistical analysis are identical, except for the number and type of aliquots submitted to chromatographic analyses, as described below.

### 2.1 Vineyard characteristics

For this study, an experimental vineyard (Quinta de Nossa Senhora de Lourdes), in which vines showing symptoms of esca complex disease are widespread, was used to develop the protocol for sampling. The 0.27-ha vineyard is located in the municipality of Vila Real in the Douro wine-growing region of Portugal, faces a north-west exposure (41° 17.12′ 31′′ N, 7° 44.07′ 22′′ W), and extends to 465 m in altitude with a slope of 3%. In the area, the mean annual air temperature is 13.97 °C, reaching the highest average of 20.87 °C during the expression of foliar symptoms of grapevine trunk diseases between June and August. Only 6% of the mean annual precipitation of approximately 1131 mm falls between June and August. The average relative humidity is 75%, and the vines receive a light intensity of 1350 µmol/m^2^/s during a 16 h photoperiod. Planted in 1995 on an Anthrosol (62% sand, 25% silt, and 13% clay; pH 4.2), the vineyard consists of 22 rows of 1247 vines of the variety Malvasia-Fina (white grape) grafted on the 196-17-Castel rootstock. The distance between the rows is 1.80 and 1.12 m separates the vines in a row.

### 2.2 Sampling procedure

The vine plants used for collecting samples in this study were selected based on their observed health status between 2014 and 2019. A plot of four rows, including 243 vines grown according to the royal-type trellis system, was delimited in the upper section of the vineyard, and all the vines in it were monitored for wood and foliar symptoms of the esca complex disease.

Leaf observations focused on identification of apoplectic symptoms (sudden wilting of shoots or leaves) and tiger stripe symptoms (chlorotic leaves that become scorched and later assume a tiger stripe-like pattern) [9], [10], [11], [12], [13]. As foliar symptoms may fluctuate from year to year [14], leaf symptoms were visually recorded every September for six years

Vines infected with esca-associated fungi in the wood were identified by extracting wood cores from the trunk using a sterilized increment borer. It was ensured that the cores represented a view as similar as possible to that observed on a horizontal cross section of the trunk. This involved taking wood cores of ca. 5 mm in diameter and ca. 80 mm long at two levels: 20 cm above the ground and 10 cm below the head of the trunk. The necrotic wood was assessed visually for the “white rot” and “brown wood streak” symptoms of the esca complex disease [15,16]. The presence of the fungi *Phaeomoniella chlamydospora, Phaeoacremonium minimum*, and *Fomitiporia mediterranea* was confirmed in diseased wood cores by recovering these fungi in culture on potato dextrose agar [12].

During the six-year period, patches of healthy (CTL), asymptomatic (ASY), and symptomatic (SY) vines were visually identified. Healthy grapevines were selected among those that did not show any esca complex disease leaf symptoms and were mostly devoid of necrotic wood tissues. Plants with brown wood streak symptoms that did not exhibit tiger stripe-like foliar symptoms for more than 50% of the study years, including in the last inspection year, were selected as asymptomatic vines. All vines that had been wood- (brown wood streak symptoms) and foliar-symptomatic (tiger stripe-like symptoms) in all years of the monitoring period were located, and two groups of plants of varying severity were defined: (i) vines that exhibited initial symptoms of leaf stripe, including small chloroses and marginal necrosis (SY1); and (ii) vines that developed moderate symptoms of leaf stripe, such as scorched spots (SY2). In the sampling year, all the vines were followed starting in June, but only those for which symptom onset occurred in the same week were used for the study. This model provided the same symptom age background for all sampled leaves and allowed the comparison of materials with homogeneous characteristics, although different in health status throughout maturation.

### 2.3 Leaf sample collection

When the grapes were ready for harvesting in early September of the last year of the inspection period, leaves were collected from different vines and their water content was determined using the oven drying method [17]. Six plants with approximately 65% leaf water content per vine group (CTL, ASY, SY1, and SY2) were selected in different rows and positions in the experimental plot to sample foliage for metabolomic analyses. Ten randomly selected leaves were harvested from the two sides of the shoot for each vine in the morning. The detached leaves were immediately frozen with liquid nitrogen and stored in a freezer at -80 °C. Leaves without visible symptoms were collected from CTL and ASY, whereas leaves with symptoms were collected from SY1 and SY2.

### 2.4 Metabolite extraction

For extraction, separation, and identification of metabolites, the method described in [18] and modified in [19] was used with some minor changes. On the day of extraction, leaf samples were lyophilized and ground using a blender (Electrodomesticos Taurus, Aromatic Ver-II, Barcelona, Spain). Metabolites were extracted from samples using an automated liquid handling robot (MicroLab STAR®, Hamilton Robotics, Inc., Reno, NV, USA), wherein 400 µL of methanol (Sigma–Aldrich; St. Louis, MO, US) was added to 20 mg of the sample. Methanol was spiked with 15 µL of four recovery standards (DL-2-fluorophenylglycine, tridecanoic acid, d6-cholesterol, and 4-chlorophenylalanine) at a concentration of 100 µg/mL to monitor the extraction efficiency. These standards were carefully selected so as not to interfere with the measurement of endogenous compounds, and were all purchased from Santa Cruz Biotechnology (Dallas, TX, USA). After mixing the components in a GenoGrinder 2000 homogenizer (Glen Mills Inc., Clifton, NJ, USA) for 2 min at 22 ± 3 °C, the mixture was centrifuged (Pro Analytical CR400R, Centurion Scientific Ltd, Chichester, UK) for 5 min at 1,500 × g and 25 °C. The supernatant was collected and split into three aliquots that were evaporated on a TurboVap® speed vacuum concentrator (Zymark Corporation, Hopkinton, MA, US). The pellet was frozen at −80 °C until chromatographic analyses.

### 2.5 Aliquot preparation

Before chromatographic analyses, the three aliquots were stored overnight under a nitrogen stream at 22 ± 3 °C and then reconstituted in three different solvents: aliquot 1 in 50 µL of 0.1% formic acid in water (acidic extract; pH ∼3.5), aliquot 2 in 50 µL of 6.5 mM ammonium bicarbonate in water (basic extract; pH ∼8.0), and aliquot 3 in 50 µL of ammonium formate in water (polar extract; pH ∼10.8). All organic solvents were of analytical grade (≥ 98%) and were obtained from Sigma–Aldrich (St. Louis, MO, USA). Methanol removal from the reconstitution solvents was found to improve the chromatographic resolution and peak shape of various early eluting compounds, thus improving their detection [18], [20].

All reconstitution solvents were spiked with a cocktail of 13 internal standards (d7-glucose, fluorophenylglycine, d3-methionine, d4-tyrosine, d3-leucine, d8-phenylalanine, d5-tryptophan, d5-hippuric acid, Cl-phenylalanine, Br-phenylalanine, d5-indole acetate, d9-progesterone, and d4-dioctylphthalate), which were used to monitor instrument performance and served as retention index markers for chromatographic alignment. These internal standards were isotopically labeled metabolites purchased from C/D/N Isotopes (Pointe-Claire, Quebec, Canada) and Santa Cruz Biotechnology (Dallas, TX, USA) and were chosen for their elution behavior, specifically their retention times and stabilities. In this case, they eluted approximately every 30 s of chromatography.

### 2.6 UHPLC (Ultrahigh performance liquid chromatography) analysis

The three aliquots were analyzed on the same day using an ACQUITY UHPLC System (Waters Corporation, Milford, MA, USA). A 5 µL aliquot of each sample was automatically injected into the UHPLC system using a 2 × needle loop overfill. A pool of aliquots generated by combining a small portion of each of the 24 grapevine samples was also injected into the UHPLC per aliquot type. The combined aliquots served as technical replicates throughout the injections to assess the overall process and platform variability. In addition, water samples were injected to serve as a baseline reference signal and to identify artifacts (water blanks). Aliquots of methanol were also injected and used to segregate contamination sources in the extraction (solvent blanks). Along with the technical replicate samples, the water and solvent blanks were spaced evenly among the experimental samples and analyzed every six injections.

The acidic extract (aliquot 1, corresponding to fraction 1 in Goufo and Cortez [8]) was first separated on an acid dedicated 2.1 × 100 mm, 1.7 µm particle size BEH C18 reverse-phase chromatographic column (Waters Corporation, Milford, MA, US) held at 40 °C. The acidic extract was gradient-eluted at 350 µL/min using (A) 0.1% formic acid and 0.05% perfluoropentanoic acid in water and (B) 0.1% formic acid and 0.05% perfluoropentanoic acid in methanol (0–70% B in 4 min, 70–98% B in 0.5 min, and 98% B for 0.9 min) and monitored for positive ions as described below. The basic extract (aliquot 2, corresponding to fraction 3 in Goufo and Cortez [8]) was monitored for negative ions in independent injections, using a separate base dedicated 2.1 × 100 mm, 1.7 µm particle size BEH C18 reverse-phase chromatographic column (Waters Corporation, Milford, MA, USA) heated to 40 °C. The basic extract was gradient-eluted at 350 µL/min using (A) 6.5 mM ammonium bicarbonate in water (pH 8) and (B) 6.5 mM ammonium bicarbonate in 95/5 methanol/water (0–70% B in 4 min, 70–98% B in 0.5 min, and 98% B for 0.9 min). The polar extract (aliquot 3, corresponding to fraction 4 in Goufo and Cortez [8]) was separated using the same UHPLC system, which was equipped with a 2.1 × 150 mm, 1.7 µm inner diameter BEH Amide Hydrophilic Interaction Liquid Chromatographic (HILIC) column (Waters Corporation, Milford, MA, US) held at 40 °C. These samples were gradient-eluted at 350 µL/min, using (A) 10 mM ammonium formate in 15% water, 5% methanol, and 80% acetonitrile; effective pH adjusted to 10.8 by adding ammonium hydroxide; and (B) 10 mM ammonium formate in 50% water and 50% acetonitrile; effective pH adjusted to 10.6 by adding ammonium hydroxide (0% B for 4 min, 0–50% B in 2 min, 50–80% B in 5 min, 80–100% B in 1 min, and 100% B for 2 min). LC-MS grade solvents were purchased from Sigma–Aldrich (St. Louis, MO, USA). Water was obtained from a Millipore Ultra-high-purity water dispenser (Billerica, MA, USA).

### 2.7 Mass spectrometry (MS) analysis

The separated metabolites were analyzed using a Q-Exactive Hybrid Quadrupole-Orbitrap high resolution/accurate mass spectrometer interfaced with a heated electrospray ionization (HESI-II) source (Thermo Fisher Scientific, Waltham, MA, US). The MS interface capillary was maintained at 350 °C, and the corona discharge current was set to 5 µA. Nebulization was performed with nitrogen, with a sheath gas flow and auxiliary gas flow of 40 and 5 arbitrary units, respectively, for both positive and negative injections. The spray voltage for positive and negative ion injection was 4.5 and 3.75 kV, respectively. The nebulizer temperature was set at 400 °C. The positive ion injection was run under acidic conditions (aliquot 1) and permitted the detection of hydrophilic compounds, including acids and phosphorylated and sulfated compounds. The negative ion injection was performed under basic (aliquot 2) and polar (aliquot 3) conditions and allowed the detection of those molecules preferring to form negative ions, including not just those compounds containing basic amines but all Brønsted-Lowry bases.

The spectrometer instrument scanned 70-1000 m/z with small variations depending on the aliquot analyzed and alternated between MS (full-scan) and MS/MS (all-ion fragmentation-scan) scans over a total instrument analysis time of 12 min, with the mass resolving power set to 50,000 (measured at 200 m/z). The scan speed was approximately nine scans per second (three MS and six MS/MS scans). The MS scan had an ion-trap target of 2 × 104 (arbitrary units) and an ion-trap fill time cutoff of 100 ms. The MS/MS normalized collision energy was set to 40, activation at Q 0.25, and activation time at 30 ms, with a 3 m/z isolation window. After an MS/MS scan of a specific m/z was obtained, the m/z was placed on a temporary MS/MS exclude list for 3.5 s. This process, called dynamic exclusion, allowed greater MS/MS coverage of the ions present in the MS scan because the instrument will not trigger an MS/MS scan of the same ion repeatedly.

### 2.8 Peak detection, integration, and data preprocessing

Xcalibur QuanBrowser 3.0. was used to acquire peak signals, including area-under-the-curve values, retention times, and mass-to-charge ratios (m/z). Prior to peak selection, six different curation procedures were performed to ensure that the peaks representing system artifacts, background noise, redundancy, and misassignments were removed, and only true chemical entities were retained.

The relative standard deviations (RSDs) of the peak area were determined for each recovery standard to confirm an average extraction efficiency of approximately 95%. Instrument variability was determined by calculating the median RSD for the 13 internal standards that were added to each sample prior to injection into the UHPLC and was found to be 7%. Overall chromatography performance was assessed by calculating the median RSD (8%) for all endogenous metabolites that were detected in 100% of the technical replicate samples. All RSD values were within the range of 10%, indicating an excellent operation of the methodology. The mass accuracy was calculated assuming that all internal standards had a < 5 ppm mass error. Peaks present in the biological samples at less than three times the levels in the water or solvent blanks were considered baseline noise or artifacts and removed from the data set. Finally, a minimum signal-to-noise ratio of 5 and a minimum width of 12 s were defined for the subtraction of other chemical noises such as peaks with poor shape or whose area trended systematically upward or downward. Data for the peaks passing the abovementioned threshold criteria were extracted and assembled into a file for further analysis.

### 2.9 Peak identification and quantification

Metabolon's Laboratory Information Management System (LIMS) was used to identify the MS peaks. The 13 internal standards were used to calculate a fixed RI value for each MS peak, which allowed sample alignment by checking the consistency of the integration of peaks sample by sample. The RI of each metabolite was obtained by logarithmic interpolation, relating the adjusted retention time to the adjusted retention times of two standards eluted before and after the peak of the metabolite.

Chemical identity was assessed by comparing the RI, accurate masses, and spectral data for each peak to those of ca. 4500 authentic standard compounds present in the LIMS library, including the adducts (e.g., K and Na), insource fragment ions, and multimers (e.g., (2m+H)^+^), generated by the standards in both negative and positive ion modes for a total of ca. 10,000 recorded MS/MS spectra. The library allowed a high-confidence identification of the detected peaks without the need for additional analyses, based on a multiparameter match: RI values within 75 RI units (∼10 s), accurate mass within 10 ppm (∼0.005 m/z), and MS/MS forward and reverse scores above 80%. A compound had to match the three independent parameters to be identified with certainty. All ions that were not assigned to any library entry across a set of injections (whether or not they met the three criteria) were not included in the present study. In the case where the same metabolite was detected in the acidic, basic, and polar aliquots, only one aliquot was chosen to represent the metabolite to avoid redundant data.

Peak areas were extracted and used as raw data for the quantitative chromatographic estimation. Raw data were adjusted by dividing out any sample weight differences to account for systematic variation in metabolite levels due to differences in the amount of starting material in each sample. The adjusted raw data were rescaled by dividing each sample value by the median value for the metabolite to obtain the scaled imputed data. The missing value for a given metabolite was assigned the minimum observed value for that metabolite, based on the assumption that the missing value was below the detection limit.

### 2.10 Statistical methods

The data were subjected to significance tests, which were performed in Array Studio 10.0 (OmicSoft Corporation, Cary, NC, US) on log10 transformed data. Mean values for each leaf group (CTL, ASY, SY1, and SY2) were calculated, and the fold change for each metabolite was determined as the ratio of the level in one group compared with that in the other group, for six pairwise comparisons. Welch's two-sample t-test was used to identify metabolites whose mean values differed between the experimental groups. This version of the two-sample t-test allows for unequal variances and tests whether the means are different, as opposed to testing whether one mean is greater than the other. For statistical significance testing, mean values were declared different for *P*-values < 0.10 and highly different for *P*-values ≤ 0.05. The false discovery rate method was used to correct for multiple testing and to account for false positives. For this, the data were first sorted by the *P*-value, a cutoff for significance of *P* ≤ 0.05, was chosen, and the q-value was calculated to give an estimate of the proportion of false discoveries for the list of compounds whose *p*-value was below the cutoff for significance. A q-value of < 0.10 is an indication of high confidence in a result.

## Ethics Statements

This article does not contain any studies involving animals or humans performed by any of the authors.

## CRediT Author Statement

**Piebiep Goufo:** Conceptualization, Methodology, Validation, Formal analysis, Investigation, Data curation, Writing – original draft, Writing – review & editing, Visualization; **Isabel Cortez:** Conceptualization, Methodology, Validation, Resources, Writing – review & editing, Supervision, Project administration, Funding acquisition.

## Declaration of Competing Interest

The authors declare that they have no known competing financial interests or personal relationships that could have appeared to influence the work reported in this paper.
